# Clean mucosal area detection of gastroenterologists versus artificial intelligence in small bowel capsule endoscopy

**DOI:** 10.1097/MD.0000000000032883

**Published:** 2023-02-10

**Authors:** Jeongwoo Ju, Hyun Sook Oh, Yeoun Joo Lee, Heechul Jung, Jong-Hyuck Lee, Ben Kang, Sujin Choi, Ji Hyun Kim, Kyeong Ok Kim, Yun Jin Chung

**Affiliations:** a Captos Co., Ltd., Yangsan, Korea; b Department of Applied Statistics, School of Social Science, Gachon University, Seongnam, Korea; c Department of Pediatrics, Pusan National University School of Medicine, Pusan National University Yangsan Hospital, Yangsan, Korea; d Department of Artificial Intelligence, Kyungpook National University, Daegu, Korea; e Department of Pediatrics, Kyungpook National University School of Medicine, Kyungpook National University Chilgok Hospital, Daegu, Korea; f Department of Internal Medicine, Kangwon National University School of Medicine, Kangwon National University Hospital, Chuncheon, Korea; g Department of Internal Medicine, Yeungnam University College of Medicine, Yeungnam University Medical Center, Daegu, Korea; h Department of Internal Medicine, Kyungpook National University Chilgok Hospital, Daegu, Korea.

**Keywords:** artificial intelligence, bowel cleanness, capsule endoscopy, small bowel

## Abstract

Studies comparing the detection of clean mucosal areas in capsule endoscopy (CE) using human judgment versus artificial intelligence (AI) are rare. This study statistically analyzed gastroenterologist judgments and AI results. Three hundred CE video clips (100 patients) were prepared. Five gastroenterologists classified the video clips into 3 groups (≥75% [high], 50%–75% [middle], and < 50% [low]) according to their subjective judgment of cleanliness. Visualization scores were calculated using an AI algorithm based on the predicted visible area, and the 5 gastroenterologists’ judgments and AI results were compared. The 5 gastroenterologists evaluated CE clip video quality as “high” in 10.7% to 36.7% and as “low” in 28.7% to 60.3% and 29.7% of cases, respectively. The AI evaluated CE clip video quality as “high” in 27.7% and as “low” in 29.7% of cases. Repeated-measures analysis of variance (ANOVA) revealed significant differences in the 6 evaluation indicators (5 gastroenterologists and 1 AI) (*P* < .001). Among the 300 judgments, 90 (30%) were consistent with 5 gastroenterologists’ judgments, and 82 (91.1%) agreed with the AI judgments. The “high” and “low” judgments of the gastroenterologists and AI agreed in 95.0% and 94.9% of cases, respectively. Bonferroni’s multiple comparison test showed no significant difference between 3 gastroenterologists and AI (*P* = .0961, *P* = 1.0000, and *P* = .0676, respectively) but a significant difference between the other 2 with AI (*P* < .0001). When evaluating CE images for cleanliness, the judgments of 5 gastroenterologists were relatively diverse. The AI produced a relatively universal judgment that was consistent with the gastroenterologists’ judgements.

## 1. Introduction

Evaluation of the visible area on endoscopy indirectly helps confirm endoscopy success. Assessing the visible area can help clinicians understand how well a lesion may be detected or missed.^[1]^ Insufficient bowel preparation increases the probability of missing polyps in the colon during colonoscopy^[1,2]^ and is an indicator that endoscopy should be repeated within a short period.^[2]^ Capsule endoscopy (CE) is a passive examination in which the doctor cannot manipulate the field of view and the information obtained is greatly affected by the visible area. Therefore, evaluating the visible area on CE is necessary to determining procedural success.^[3–5]^

Most indicators for bowel cleanliness are limited to colonoscopies. However, these judgments tend to be subjective. During colonoscopy, when a gastroenterologist evaluates bowel preparation quality, many instruments, such as the aronchick bowel preparation scale, Boston bowel preparation scale, Ottawa bowel preparation scale, and Chicago bowel preparation scale, are used to reach an objective judgment,^[6,7]^ however, those are still subjective. With the Boston bowel preparation scale, gastrointestinal endoscopists familiarize themselves with photographs of objective indicators of varying degrees of bowel cleanliness and attempt to perform an objective evaluation.^[8]^ However, even if gastroenterologists attempt to establish objective standards, interobserver reliability is diverse and judgments may differ among evaluators.^[6,7]^ Moreover, there has been much less research in the field of CE. Evaluation of the intestinal preparation in CE is mainly performed by setting the visible range of the mucosa.^[3–5,9,10]^ However, few studies have examined the consistency of judgments among CE readers.

Accordingly, many quantitative evaluations using artificial intelligence (AI) have been performed to evaluate the visible region as objectively as possible. The authors of a previous study reported the accuracy of clean mucosal prediction as 95.7% by the AI algorithm using the visible region dataset by the semantic segmentation method.^[11]^ Although another study evaluated the accuracy of each frame, video CE accuracy data remain limited. We updated the algorithm by adding more diverse images to the existing dataset and established a system that can be used to detect areas of clean mucosa on video CE images.

Dynamic imaging, such as CE video, makes it nearly impossible to create ground truths that AI uses as a basis for analysis. Therefore, this study aimed to compare our algorithm with the clinical judgment of 5 gastroenterologists. First, we first analyzed the judgment consistency among CE readers. Second, we compared the judgments of the 5 gastroenterologists with the AI results.

## 2. Methods

### 2.1. Image selection and study design

Capsule endoscopic images of 100 patients obtained from the 2 hospitals were used. The patients baseline demographic data were also collected.

**Figure 1.**
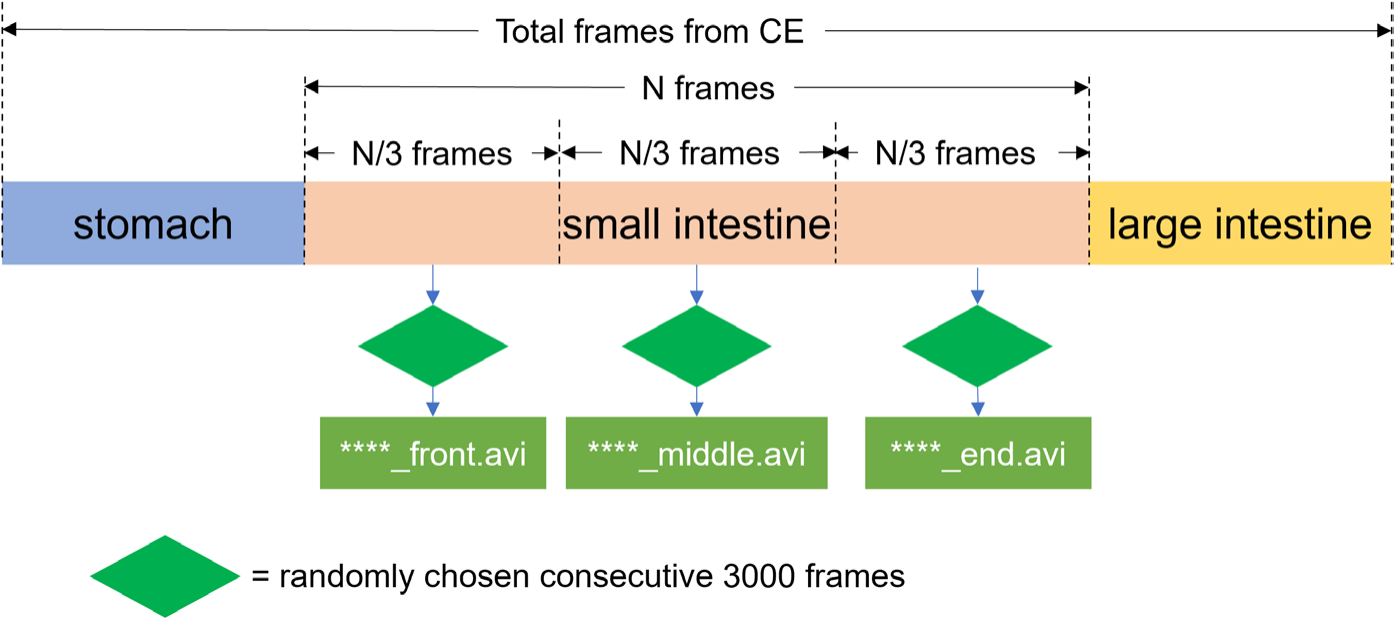
Schema of capsule endoscopy video selection method.

The small intestine was divided into 3 sections to facilitate image processing. To extract various images from different sections, 3000 consecutive frames were randomly extracted from each third and produced as a video (Fig. 1). A total of 300 video clips were generated. The video was produced in *.avi format so that it could be played while limiting the speed to < 30 frames per second. Because the images were randomly generated, several CE videos did not move and remained in a fixed position in the small intestine. Hence, to identify how CE stasis affects judgment discrepancies between AI and gastroenterologists, we calculated the pixel-level difference for each video to roughly measure the capsule movement speed.

Five gastrointestinal endoscopy specialists (G5, > 100 capsules read; G1, > 200 capsule read; G2, G3, G4, > 500 capsule read) in South Korea working in high-volume tertiary hospitals analyzed the 300 CE video clip images and evaluated the visible areas. G5 had 2 years, G1 had 5 years, G3 had 10 years, G2 had 15 years, and G4 had 16 years of experience reading CE videos. Based on the ratio of visible area, image quality was classified as 100% to 75% (high), 75% to 50% (middle), or < 50% (low).

The clean mucosal detection range for the 300 videos was also evaluated using the AI algorithm. Depending on the visible area of each frame, the least visible area was assigned a value of 0 and the entire visible area was assigned a value of 1. The average was calculated and assumed to be the result equivalent to the visualization scale (VS; refer to Equation 1 in a previous study^[11]^). Additionally, according to the range of the evaluated average visible area for comparison with the gastroenterologists judgment, the results were scored 1.0 to 0.75 (high), 0.75 to 0.50 (middle), and < 0.50 (low).

### 2.2. Algorithm outline

Ju et al^[11]^ established a semantic segmentation dataset to capture clean mucosal areas on CE images. Their dataset comprised CE studies (PillCam SB3; Medtronic, Minneapolis, MN), with 10,033 frames captured from the videos of 179 patients. By adding 333 CE studies (MiroCam MC1600; IntroMedic, Seoul, Korea) to the existing dataset, we generated an additional dataset that was used to train and test the AI algorithm developed based on DeepLab v3.^[12]^ Overall, 2319 samples from 12 patients (10 patients for PillCam + 2 patients for MiroCam) and 10,914 samples from 500 patients (169 patients for PillCam + 331 patients for MiroCam) were used for the testing and training processes, respectively. We trained DeepLab v3, whose backbone model was ResNet50^[13]^ with a batch size of 64, 3 classes (clean, dark, and floats/bubbles), and pixel-wise cross-entropy loss. The following equation (Equation 1) was used to predict the visible area, in which M denotes the total number of frames from patients, size (image) is the pixel size of a given image, $C_{i}$, $D_{i}$, and $F_{i}$ are the predicted set of pixels that belong to the clean, dark, and floats/bubble classes for the i-th image, and |·| denotes the cardinality of the set.


$$\begin{array}{l} VS=\;\;\;\frac{1}{M}\underset{i=1}{\overset{M}{\sum }}\;\frac{\left|C_{i}\right|}{size\left(image\right)}=\;\;\;\frac{1}{M}\underset{i=1}{\overset{M}{\sum }}\;\frac{size\left(image\right)-\left|D_{i}\overset{}{\cup }F_{i}\right|}{size\left(image\right)},\;\;\;\;\;0\leq VS\leq 1 \end{array}$$ (1)

The achieved testing performance was 0.8134 for the MiroCam and 0.7801 for the Pillcam in terms of the mean interception over union. Specifically, the clean area prediction accuracies were 95.9% and 93.3% for the MiroCam and PillCam datasets, respectively.

### 2.3. Statistical analysis

For the nominal scale, the frequency was calculated; for the continuous variable, the mean and standard deviation were calculated and compared using a *t* test for independent variables. Spearman correlation coefficient was used to analyze the correlations between continuous variables.

To compare the VS results, a repeated-measures ANOVA was performed to verify the significance of the difference between the 5 human evaluators and 1 AI evaluator that measured each image from the dataset. The evaluation indices of high, middle, and low were quantified as 3, 2, and 1, respectively, and analyzed.

Furthermore, we quantified the VS that the AI engine yielded as follows: above 0.75 (high), between 0.5 and 0.75 (middle), and below 0.5 (low) were assigned the values of 3, 2, and 1, respectively.

As a post hoc analysis, a pairwise comparative analysis was performed of the 6 evaluation indicators using Bonferroni’s method. The agreement between the 5 gastroenterologists and 1 AI evaluator was analyzed using histograms. All statistical verifications were performed based on a significance level of 0.05, and the statistical analyses were performed using R statistical software (version 4.0.5; R Foundation for Statistical Computing, Vienna, Austria).

### 2.4. Ethics statement

This study was approved by the Institutional Review Boards (IRB) of Pusan National University Yangsan Hospital (IRB no. 05-2021-070) and Kyungpook National University Chilgok Hospital (IRB no. 2021-07-031), which waived the requirement for informed consent owing to the study’s retrospective nature.

## 3. Results

### 3.1. Basic characteristics of 100 CE videos (Table 1)

Among the 100 patients, the mean age was 54.8 ± 20.0 years and 65 were male. The main reasons for CE were work-up for gastrointestinal bleeding in 51 patients, diagnosis of small intestinal lesions in 33 patients, and obscure gastrointestinal bleeding in 16 patients. Positive findings were noted in 66 patients, and 24 had Crohn disease (CD). The mean stomach transit time was 0:46:18 and the mean small bowel transit time was 6:13:03. Among the 24 patients with CD, the mean age was 53.1 ± 21.5 years, similar to that of the non-CD patients (n = 76, 55.3 ± 0.17 years), while the mean small bowel transit time was 5:12:23, significantly shorter than that of the others (n = 76, 6:32:12, *P* = .005).

**Table 1:** Patients demographic and clinical data.

Age (yr)	Mean	54.8 ± 20.0		Median	58.7 (range: 12.8–94.5)
	Male	52.0 ± 20.4		Female	58.3 ± 29.0
Sex	Male: Female			65: 35	
Reason for capsule endoscopy	Gastrointestinal bleeding				51
	Diagnosis of small intestinal lesion				33
	Obscure GI bleeding				16
Diagnosis/Findings after capsule endoscopy	Crohn disease				24
	Polyp(s)				5
	Vascular lesion				5
	Other findings				32
	Normal				34
Stomach transit time	0:46:18			Range	0:00:09–3:47:15
Small bowel transit time	6:13:03			Range	1:35:16–10:52:57

GI = gastrointestinal.

### 3.2. Results of gastroenterologist evaluations

Among the 300 images, 90 (30.0%) showed congruent results for all 5 gastroenterologists, 79 (26.3%) showed the same results for 4 gastroenterologists, and 110 (36.7%) showed the same results for 3 gastroenterologists. Overall, 279 (93.0%) showed the same results for more than 3 gastroenterologists, while 21 (7.0%) showed confusing results for the 5 gastroenterologists.

Among the 5 gastroenterologists, 3 (G2, G3, and G4) had more than 10 years experience reading CE videos. Among the 3 more experienced gastroenterologists, 120 images (40.0%) showed congruent results, while 165 (55.0%) showed congruent results for 2 gastroenterologists. Fifteen images (5.0%) showed different results for all 3 experts.

**Figure 2.**
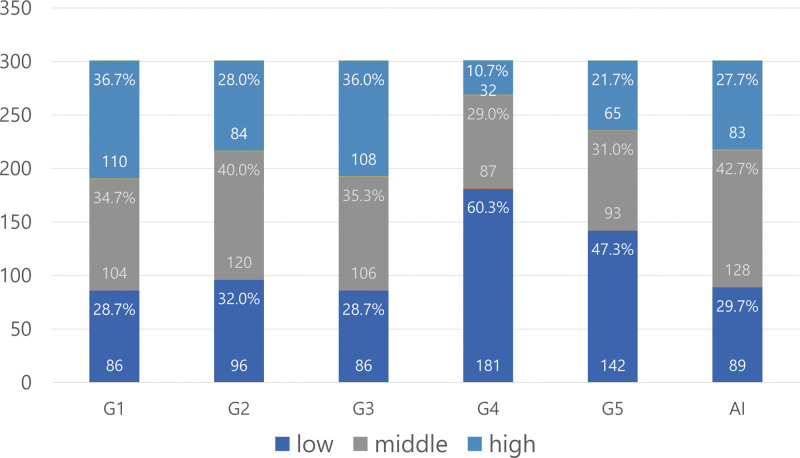
Distribution of visible area evaluations of 300 capsule endoscopy videos of the 5 gastroenterologists and 1 artificial intelligence (AI) system. AI = artificial intelligence.

G1 and G3 exhibited the highest rate of assigning a high value to the video clips (36.7% and 36.0%, respectively). The other gastroenterologists (G2, G4, and G5) assigned a high value to the video clips at rates of 28.0%, 10.7%, and 21.7% (Fig. 2), respectively. The middle value rates for G1, G2, and G3 were 34.7% to 40%, while those for G4 and G5 were 29.0% to 31.0%, showing no significant difference. G1, G2, and G3 showed low value rates of 28.7% to 32.0%, while G4 and G5 assigned the highest low value rates of 60.3% and 47.3%, respectively. Bonferroni’s multiple comparison test (Table 2) showed no significant difference between G1 and G3 (*P* = 1.0000) or between G1 and G2 (*P* = .0141); however, G2 and G3 (*P* = .0027) differed significantly, as did G4 and G5 (*P* < .0001).

**Table 2:** Evaluations by gastroenterologists versus artificial intelligence engine.

Gastroenterologists	Mean CE Quality Index	Standard deviation	Comparison between Gastroenterologists and AI (*P* value)				
			1	2	3	4	5
Gastroenterologist 1	2.08	0.806		.0141	1.0000	<.0001	<.0001
Gastroenterologist 2	1.96	0.775			.0027	<.0001	<.0001
Gastroenterologist 3	2.07	0.802				<.0001	<.0001
Gastroenterologist 4	1.50	0.682					<.0001
Gastroenterologist 5	1.74	0.791					
Artificial intelligence	1.98	0.758	.0961	1.0000	.0676	<.0001	<.0001

AI = artificial intelligence, CE = capsule endoscopy.

### 3.3. AI evaluation results

The AI considered 27.7% of the total video clips as having high value. The AI assigned a middle value to 42.7%, a slightly higher rate than that assigned by all 5 gastroenterologists. The AI considered 29.7% of the videos as having low value.

### 3.4. Judgment of 5 gastroenterologists versus 1 AI system

AI considered 27.7% of the videos as having high value, a rate that was nearly identical to that of G2 (28.0%), whose rate was the median of the 5 gastroenterologists. The low value rate for AI was 29.7%, which placed it among G1, G3, and G2. When the decisions (high, middle, and low) were quantified as 3, 2, and 1, respectively, the average values of the indices assigned by G1 and G3 were the highest at 2.08 and 2.07, respectively, while those of G2, G4, and G5 were 1.96, 1.50, and 1.74, respectively (Table 2). The average index assigned by the AI was 1.98, similar to those of G1, G2, and G3 (Table 2).

In Bonferroni’s multiple comparison test (Table 2), AI showed no significant difference from G1, G2, and G3 (*P* = .0961, *P* = 1.0000, and *P* = .0676, respectively), whereas G4 and G5 showed a significant difference (*P* < .0001). Repeated-measures ANOVA revealed a significant difference among the 6 evaluation indicators for each video (*P* < .001).

**Figure 3.**
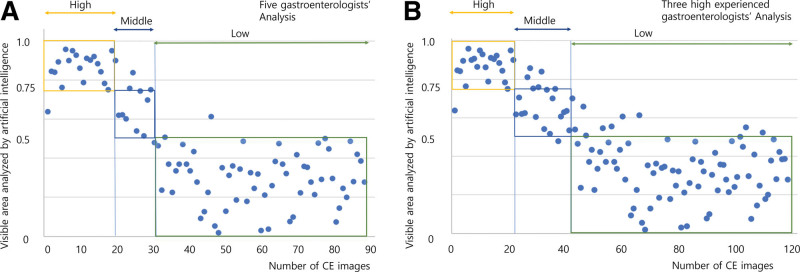
Histograms of 90 capsule endoscopy (CE) video clips in which the judgments of 5 gastroenterologists coincided (A) and 120 CE video clips in which the judgments of the 3 most experienced gastroenterologists coincided (B). The horizontal axis of the figure represents the artificial intelligence judgment of 0–1. CE = capsule endoscopy.

To examine the agreement/disagreement pattern between AI and the gastroenterologists, we used 90 video clips for which all gastroenterologists offered the same evaluation results. Among the 90 video clips, 20 (22.22%) were graded as high (yellow box in Fig. 3), 11 (12.22%) as middle (blue box in Fig. 3), and 59 (65.56%) as low (green box in Fig. 3). All gastroenterologists seldom agreed with the middle decision, whereas they reached high agreement with the low decision. Among the 90 video clips, the judgment of 82 cases (91.11%) was consistent with the AI evaluation (Fig. 3A). The gastroenterologist’s high or low evaluation agreement with AI was 95.0% and 94.9%, respectively. In contrast, when the gastroenterologist’s evaluation was middle, the degree of agreement decreased (64.6%). Furthermore, among the 11 video clips the AI evaluated as having a middle value, 1, 7, and 3 video clips were assigned a value of high, middle, and low by the gastroenterologists, respectively. The corresponding mean AI scores for video clips 1, 7 and 3 were 0.638, 0.621, and 0.541, respectively. Hence, although all AI evaluations were middle for those 11 video clips, the mean score decreased consistent with the gastroenterologists’ judgment (Table 3).

**Table 3:** Mean AI score for 90 videos for which the 5 gastroenterologists’ ratings were consistent.

		AI										
		High			Middle				Low			Total
		Number	Mean AI Score	SD	Number	Mean AI Score	SD	Number		Mean AI Score	SD	
**Gastro-enterologists**	**High**	19	0.873	0.064	1	0.638						20
	**Middle**	3	0.783	0.049	7	0.621	0.081	1		0.480		11
	**Low**				3	0.541	0.063	56		0.283	0.130	59
	**Total**	22	0.860	0.069	11	0.601	0.079	57		0.286	0.131	90

AI = artificial intelligence, SD = standard deviation.

For the 120 images that the 3 most experienced gastroenterologists (G2, G3, and G4) showed congruency, the overall agreement with AI was 86.7%, while those for high, middle, and low was 95.5%, 66.7%, and 89.6%, respectively (Fig. 3B).

The pixel-level difference was negatively correlated with the differences between the decisions of gastroenterologists and AI, although the statistical significance was weak (Spearman r = -0.148, *P* = .010). In other words, the less the difference in the images (CE stasis), the higher the discrepancies between the decisions made by the gastroenterologists and the AI.

## 4. Discussion

Identification of the visible area on CE does not directly affect a patient’s diagnosis. Along with recording duration, judgment of the visible region is a very important indicator of intra-procedural quality on CE.^[14,15]^ Visible area analysis helps clinicians analyze examination quality and the likelihood of missing the lesion according to bowel preparation degree. Viazis et al^[3]^ reported that lesions were found more than twice in the group with a high versus low visible area on CE. Moreover, these analyses can be performed together with the clinical situation for bowel preparation,^[9,10]^ which can help improve bowel preparation in the future. In fact, many studies have analyzed the visible region on CE to analyze the methodology used to improve bowel preperation.^[16]^ However, most human-readable CE images are analyzed using dozens or hundreds of videos or multiple readers.^[16]^ There are many variables in the study, but the interobserver differentiation between CE readers is unclear. This problem can be solved if a large-scale image can be analyzed homogeneously. This can help develop the best binding results and CE protocol.

Our study revealed that gastroenterologists’ judgments were diverse and statistically different. It was also determined that the gastroenterologists’ experiments did not significantly influence the visualization score. In particular, in studies that divide grades, a decision in the middle rather than either extreme is very confusing.^[17]^ Moreover, if the doctor’s decision is different, the expertise decision is followed.^[17]^ There is reportedly no significant difference between experts and beginners when a certain portion of training is performed in the evaluation of bowel cleanliness.^[18]^

To analyze images similar to the actual images, we randomly extracted images without processing. In an actual CE, it often does not move and remains in a specific compartment. The AI rating of high versus the doctor rating as middle was the most common error. Among the 16/120 (13.3%) cases in which the judgments of the 3 most experienced gastroenterologists were consistent and the AI judgment differed, 14 showed higher scores for the latter than the former. Although it is difficult to assign statistical significance, most images were close to still images. The pixel-level difference was negatively correlated with those of the gastroenterologists and AI. AI mechanically evaluates all still frames equally; however, gastroenterologists add clinical judgment to this situation.

To the best of our knowledge, this is the first study to compare the judgments of 5 gastroenterologists and 1 AI regarding the visible region of CE. We prepared 300 video clips collected from 100 patients and presented them to 5 gastroenterologists and an AI engine. All 5 gastroenterologists reached the same judgments in 30% of cases. The judgments of the gastroenterologists tended to be consistent for clear images and those with small visible areas. Two gastroenterologists (G1 and G3) evaluated the visible area positively, while 2 others (G4 and G5) offered conservative judgments. There was no statistically significant difference between the AI judgments and those of G1, G2, and G3. Therefore, we concluded that the AI evaluation was not significantly different from that of humans. In the CE videos, where the visible area was judged as < 50% by the gastroenterologists, the agreement between the gastroenterologists and the AI was 94.9%. Additionally, the judgment of the AI was represented on a numerical scale other than high, middle, and low, which provided more information, and the scale seemed to match the gastroenterologists’ judgments.

The strengths of this study are as follows. First, this is the first to directly compare the judgments of gastroenterologists and AI of video CE. Second, in this study, the algorithm used and the CE video evaluated were obtained from 2 sources (PillCam and MiroCam), although no difference was noted between the 2 imaging modalities. Third, the evaluation did not depend on ground-truth data produced by a small number of people, and the training and test datasets differed. This suggests that the AI algorithm yields similar conclusions for various images.

This study has some limitations. First, there are many video capture devices on the market other than those used here; therefore, it is necessary to train and validate the algorithm to account for this aspect. The CE video clips used in this study were 3000 frames long, 3 video clips were extracted per video, and 100 videos were evaluated. Therefore, viewing them as mass data is challenging. Additionally, as AI cannot predict the movement speed of the CE device inside the small intestine, we occasionally obtained inaccurate evaluation results, particularly when the CE device remained in the same location of the small intestine and delivered a video clip comprising nearly identical images. In our study, pixel-level similarities may have influenced the AI and gastroenterologists’ judgments. This aspect requires fine tuning correction in the future.

Nevertheless, the findings of this study are valuable, as they suggest that AI can be effectively used to evaluate CE videos, especially when it is difficult for gastroenterologists to judge cases quantitatively. In the future, AI can be used for quantitative assessments, such as of disease severity. The homogeneous and quantitative judgments provided by AI could be helpful in the treatment and follow-up of various diseases.

## Author contributions

**Conceptualization:** Jeongwoo Ju, Hyun Sook Oh, Yeoun Joo Lee, Heechul Jung, Jong-Hyuck Lee.

**Data curation:** Jeongwoo Ju, Hyun Sook Oh, Yeoun Joo Lee, Heechul Jung, Jong-Hyuck Lee, Ben Kang, Sujin Choi, Ji Hyun Kim, Kyeong Ok Kim, Yun Jin Chung.

**Writing – original draft:** Jeongwoo Ju, Hyun Sook Oh, Yeoun Joo Lee, Heechul Jung, Jong-Hyuck Lee.

**Writing – review & editing:** Jeongwoo Ju, Hyun Sook Oh, Yeoun Joo Lee, Heechul Jung, Jong-Hyuck Lee.
